# Supplementary figures and images for: Chemical effects of diceCT staining protocols on fluid-preserved avian specimens
Source: PLoS One. 2020 Sep 18;15(9):e0238783. doi: 10.1371/journal.pone.0238783 (PMC7500670; doi:10.1371/journal.pone.0238783)

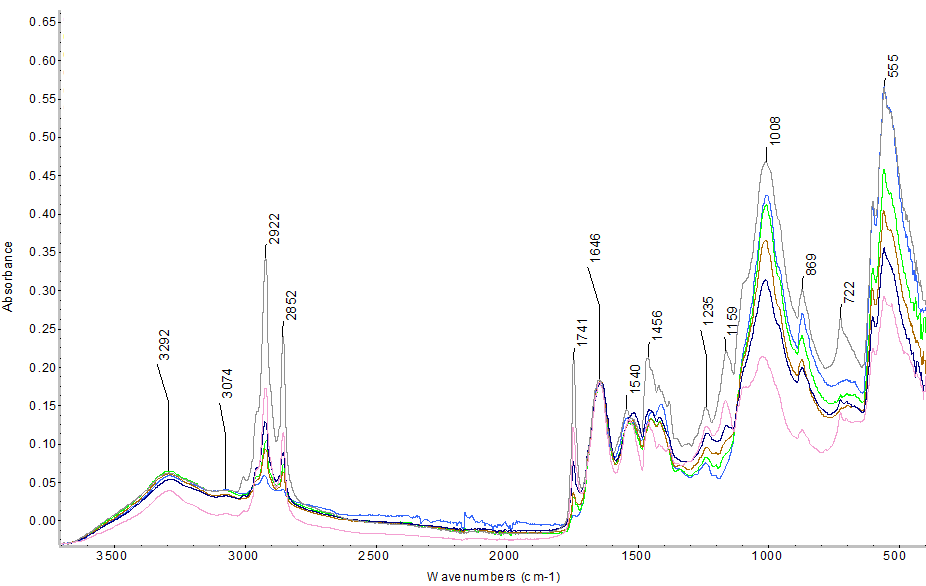

Supplement: S1 Fig — Sample ID numbers: 657966 (unstained control 1; light gray) and 657969 (unstained control 2; light blue), 657963 (stain 3; green), 657968 (stain 2; brown), 657967 (stain 4; purple), and 657965 (stain 1; pink) (normalized to the Amide I peak from collagen at 1646 cm-1). (TIF) [file pone.0238783.s001.tif]
