# Supplementary material for: Chemical effects of diceCT staining protocols on fluid-preserved avian specimens
Source: PLoS One. 2020 Sep 18;15(9):e0238783. doi: 10.1371/journal.pone.0238783 (PMC7500670; doi:10.1371/journal.pone.0238783)
Supplement: S2 Table — The six-digit number at the beginning of the sample ID corresponds to the USNM specimen number of the specimen from which the sample was taken. “A” at the end of the number indicates that the sample was taken before staining, “Z” indicates that the sample was taken after refreshing stain, and “B” indicates that the sample was taken once the specimen was fully stained. The “-M” suffix at the end of all the codes denotes muscle samples. (DOCX) [file pone.0238783.s003.docx]

**S2 Table. Muscle sample weights for all conditions.**

| Sample | Weight |
| --- | --- |
| 657963A-M | 2.9 mg |
| 657963B-M | 7.6 mg |
| 657964A-M | 2.4 mg |
| 657964Z-M | 16.5 mg |
| 657964B-M | 6.5 mg |
| 657967A-M | 7.6 mg |
| 657967A-M | 8.9 mg |
| 657968A-M | 4.8 mg |
| 657968B-M | 5.8 mg |

The six-digit number at the beginning of the sample ID corresponds to the USNM specimen number of the specimen from which the sample was taken. “A” at the end of the number indicates that the sample was taken before staining, “Z” indicates that the sample was taken after refreshing stain, and “B” indicates that the sample was taken once the specimen was fully stained. The “-M” suffix at the end of all the codes denotes muscle samples.
