# Supplementary material for: Chemical effects of diceCT staining protocols on fluid-preserved avian specimens
Source: PLoS One. 2020 Sep 18;15(9):e0238783. doi: 10.1371/journal.pone.0238783 (PMC7500670; doi:10.1371/journal.pone.0238783)
Supplement: S3 Table — (DOCX) [file pone.0238783.s004.docx]

**S3 Table. MorphoSource ARK IDs for all scans generated by this study.**

| **I2KI in 70% EtOH – USNM 657963** | |
| --- | --- |
| Weeks in Stain | MorphoSource ARK ID |
| 0 | ark:/87602/m4/M115356 |
| 1 | ark:/87602/m4/M115357 |
| 2 | ark:/87602/m4/M115360 |
| 3 | ark:/87602/m4/M115362 |
| 4 | ark:/87602/m4/M115364 |
| 5 | ark:/87602/m4/M115365 |
| **I2KI in water – USNM 657964** | |
| Weeks in Stain | MorphoSource ARK ID |
| 0 | ark:/87602/m4/M115367 |
| 2 | ark:/87602/m4/M115371 |
| 3 | ark:/87602/m4/M115375 |
| 4 | ark:/87602/m4/M115379 |
| 5 | ark:/87602/m4/M115382 |
| 6 | ark:/87602/m4/M115383 |
| 10 | ark:/87602/m4/M115386 |
| **I2KI in water – USNM 657965** | |
| Weeks in Stain | MorphoSource ARK ID |
| 3 | ark:/87602/m4/M115387 |
| 6 | ark:/87602/m4/M115388 |
| **I2 in 70% EtOH – USNM 657967** | |
| Weeks in Stain | MorphoSource ARK ID |
| 0 | ark:/87602/m4/M115389 |
| 1 | ark:/87602/m4/M115391 |
| 2 | ark:/87602/m4/M115394 |
| 3 | ark:/87602/m4/M115395 |
| 4 | ark:/87602/m4/M115396 |
| 5 | ark:/87602/m4/M115398 |
| 6 | ark:/87602/m4/M115399 |
| 7 | ark:/87602/m4/M115400 |
| 8 | ark:/87602/m4/M115401 |
| **I2 in 100% EtOH – USNM 657968** | |
| Weeks in Stain | MorphoSource ARK ID |
| 0 | ark:/87602/m4/M115402 |
| 2 | ark:/87602/m4/M115405 |
| 3 | ark:/87602/m4/M115406 |
| 4 | ark:/87602/m4/M115407 |
| 5 | ark:/87602/m4/M115408 |
| 6 | ark:/87602/m4/M115409 |
