# Supplementary material for: Chemical effects of diceCT staining protocols on fluid-preserved avian specimens
Source: PLoS One. 2020 Sep 18;15(9):e0238783. doi: 10.1371/journal.pone.0238783 (PMC7500670; doi:10.1371/journal.pone.0238783)
Supplement: S1 File — (DOCX) [file pone.0238783.s005.docx]

Supporting Information for

**Chemical and Molecular Effects of Diffusion-Based Iodine Contrast-Enhancing Stains on Fluid-Preserved Avian Specimens**

Catherine M. Early, Ashley C. Morhardt, Timothy P. Cleland, Christopher M. Milensky, Gwénaëlle Kavich, Helen F. James

# Detailed methods

## Bone protein extraction

Bone samples were weighed (S1 Table) and suspended in 600 µL of 400 mM ammonium phosphate dibasic, 200 mM ammonium bicarbonate, 4 M guanidine HCl (Cleland and Vashishth, 2015). They were homogenized for 2 cycles for 45 s at 6.95 m/s in a BeadRuptor Elite (Omni International). Protein concentration was measured using Pierce Coomassie (Bradford) Protein assay following manufacturer instructions.

## Muscle protein extraction

Muscle was mechanically separated and weighed (S2 Table), and subsamples were initially suspended in 600 µL of 50 mM ammonium bicarbonate and homogenized for one cycle at 6 m/s for 30 s. After measuring protein concentration using Bradford assay, an additional 600 µL of 1% SDS (final concentration of 0.5% SDS) was added, and the subsample was homogenized again at 4 m/s for 30 s. Protein concentration was measured using the Pierce BCA assay.

## Protein digestion

For both bone and muscle, proteins were digested using single-pot solid-phase sample preparation (SP3; Cleland, 2018; Moggridge et al., 2018). In short, 10 µg of protein was taken and reduced and alkylated with 10 mM TCEP, 40 mM chloroacetamide at 95^o^C for 10 minutes (Kulak et al., 2014). An equal volume of ethanol (sample volume + reducing agent + bead volume) and 10 µL of SeraMag Speedbeads (20 µg/µL) were added and allowed to incubate for 10 minutes at room temperature. Beads were separated on a magnet, and beads were washed three times with 80% ethanol. After washing, the beads were resuspended in 90 µL of 50 mM ammonium bicarbonate, and 0.4 µg of Promega modified trypsin was added (1:25 trypsin:protein). All samples were incubated overnight at 37^o^C and then desalted and concentrated using in house prepared Empore C18 (3M) stage tips (Rappsilber et al., 2007). Following drying under vacuum, all samples were resuspended in 0.1% formic acid at a final concentration of 0.5 µg/µL.

## Liquid chromatography-mass spectrometry (LC-MS)

For both bone and muscle, 0.5 µg of protein was injected onto an in-house packed 3 cm Thermo BioBasic C18 trap column (75 µm i.d., 4.6 µm beads) and separated on an in-house packed 21 cm Thermo BioBasic C18 analytical column with integrated emitter with an Ultimate 3000 UHPLC (ThermoScientific). Peptides were separated with the following gradient: 0−8 min 2% B, 8−98 min 2−55% B, 98−100 min 55−90% B, 100−103 min 90% B. Buffer A is 0.1% Optima-grade formic acid in Optima-grade water, and buffer B is 99.9% Optima-grade acetonitrile with 0.1% Optima-grade formic acid. Peptides were nanosprayed into a ThermoScientific LTQ Orbitrap Velos and analyzed with the following parameters: MS1: 60k resolution, 100 ms ion time, 1E6 AGC target; MS2: top 8 peaks fragmented with higher energy collisional dissociation (HCD; 30% NCE), 15k resolution, 5 m/z isolation width, 250 ms AGC, 5E5 AGC target.

## Database searching

All raw data were searched with PEAKS 8.5 against a *Taeniopygia guttata* database downloaded from Uniprot (June 20, 2019) because no *Passer domesticus* database was available. The following parameters were used with specific differences for bone marked. Data refine: default settings. De novo: parent mass tolerance 10 ppm; fragment mass tolerance 0.02 Da; enzyme trypsin; fixed modification: carbamidomethylation (C); variable modifications: deamidation (NQ), oxidation (M), oxidation or hydroxylation (bone only); max variable PTM per peptide 3; report 5 peptides. Peaks DB: parent mass tolerance 10 ppm; fragment mass tolerance 0.02 Da; enzyme trypsin; fixed modification: carbamidomethylation (C); variable modifications: deamidation (NQ), oxidation (M), hydroxylation P (bone only); database *Taeniopygia guttata*; contaminant database contaminants; FDR estimation enabled, max variable PTM per peptide 3; max mixed cleavages 2. Peaks PTM: All 313 PTMs available in PEAKS 8.5; de novo score threshold 15; peptide hit threshold 30.0; max variable PTM per peptide 5. SPIDER: L equals I true; Q equals K true.

All raw data were also searched against the same *Taeniopygia guttata* (zebra finch) database using MetaMorpheus 0.0.301 on the Smithsonian High Performance Cluster (SI/HPC). The following calibration settings were used: protease = trypsin; maximum missed cleavages = 2; minimum peptide length = 7; maximum peptide length = unspecified; initiator methionine behavior = Variable; fixed modifications = Carbamidomethyl on C, Carbamidomethyl on U; variable modifications = Oxidation on M; max mods per peptide = 2; max modification isoforms = 1024; precursor mass tolerance = ±15.0000 ppm; product mass tolerance = ±25.0000 ppm. The following G-PTM-D settings were used: protease = trypsin; maximum missed cleavages = 2; minimum peptide length = 7; maximum peptide length = unspecified; initiator methionine behavior = Variable; max modification isoforms = 1024; fixed modifications = Carbamidomethyl on C, Carbamidomethyl on U; variable modifications = Oxidation on M; G-PTM-D modifications count = 152; precursor mass tolerance(s) = 5-43.005813657,-41.026549101,-33.987721239,-32.008456684,-30.010564684,-28.031300129,-25.031634482,-18.010564684,-17.026549101,-16.042533,-15.99491462,-14.015650065,-9.03671986200001,-2.015650064,-1.031634,-0.984015583,0,0.984015583,1.968032,3.99491462,4.955394,12,14.015650064,14.999666,15.99491462,16.978931,19.989829239,21.969391633,21.98194425,22.965959,27.010899036,27.99491462,28.031300129,28.990163592,29.974179175,31.989829239,32.973845,37.946940799,37.955881454,37.976858,42.010564684,42.046950193,42.994581,43.005813656,43.963886,43.989829239,44.985078211,47.984743859,52.911461233,53.919286266,56.026214748,57.021463721,58.005479304,61.913491946,61.921772688,63.992508,68.026214748,70.041864813,71.037113785,79.956815033,79.966330889,86.000393923,87.044604465,100.016043988,101.948274,114.031694052,125.896646868,147.166337053,153.176901737,159.932662,181.208201865,203.079372521,204.187800774,210.198365458,226.077598874,229.014009359,238.229665586,251.793293736,377.689940603,541.06110975 Da; product mass tolerance = ±20.0000 ppm. The following search settings were used: protease = trypsin; maximum missed cleavages = 2; minimum peptide length = 7; maximum peptide length = unspecified; initiator methionine behavior = Variable; fixed modifications = Carbamidomethyl on C, Carbamidomethyl on U; variable modifications = Oxidation on M; max mods per peptide = 2; max modification isoforms = 1024; precursor mass tolerance = ±5.0000 ppm; product mass tolerance = ±20.0000 ppm; report PSM ambiguity = True. All peptide spectral matches (PSMs) and protein groups were filtered at a 1% false discovery rate (FDR).
